# Metatranscriptome sequencing identifies *Escherichia* are major contributors to pathogenic functions and biofilm formation in diabetes related foot osteomyelitis

**DOI:** 10.3389/fmicb.2022.956332

**Published:** 2022-08-01

**Authors:** Michael Radzieta, Matthew Malone, Mehtab Ahmad, Hugh G. Dickson, Saskia Schwarzer, Slade O. Jensen, Lawrence A. Lavery

**Affiliations:** ^1^South West Sydney Limb Preservation and Wound Research, South Western Sydney Local Health District (LHD), Sydney, NSW, Australia; ^2^Infectious Diseases and Microbiology, School of Medicine, Western Sydney University, Sydney, NSW, Australia; ^3^Department of Vascular Surgery, Liverpool Hospital, South Western Sydney Local Health District (LHD), Sydney, NSW, Australia; ^4^South Western Clinical School, University of New South Wales, Sydney, NSW, Australia; ^5^South Western Clinical School, University of New South Wales, Sydney, NSW, Australia; ^6^Department of Plastic Surgery, University of Texas Southwestern Medical Center, Dallas, TX, United States

**Keywords:** metatranscriptome, RNA-sequencing, *Escherichia*, diabetes, bone, diabetic foot osteomyelitis (DFO)

## Abstract

Osteomyelitis in the feet of persons with diabetes is clinically challenging and is associated with high rates of amputation. In this study RNA-sequencing was employed to explore microbial metatranscriptomes with a view to understand the relative activity and functions of the pathogen/s responsible for diabetes foot osteomyelitis (DFO). We obtained 25 intraoperative bone specimens from persons with confirmed DFO, observing that *Escherichia* spp. (7%), *Streptomyces* spp. (7%), *Staphylococcus* spp. (6%), *Klebsiella* spp. (5%) and *Proteus* spp. (5%) are the most active taxa on average. Data was then subset to examine functions associated with pathogenesis (virulence and toxins), biofilm formation and antimicrobial/multi-drug resistance. Analysis revealed *Escherichia* spp. are the most active taxa relative to pathogenic functions with K06218 (mRNA interferase *relE*), K03699 (membrane damaging toxin *tlyC*) and K03980 (putative peptidoglycan lipid II flippase *murJ*), K01114 (membrane damaging toxin plc) and K19168 (toxin cptA) being the most prevalent pathogenic associated transcripts. The most abundant transcripts associated with biofilm pathways included components of the biofilm EPS matrix including glycogen synthesis, cellulose synthesis, colonic acid synthesis and flagella synthesis. We further observed enrichment of a key enzyme involved in the biosynthesis of L-rhamnose (K01710 -dTDP-glucose 4,6-dehydratase *rfbB, rmlB, rffG*) which was present in all but four patients with DFO.

## Introduction

Foot infections in persons with diabetes are the amongst the most frequent causes of hospitalization and lower extremity amputation (Lavery et al., 2006). Infections commonly originate in ulcerated tissue but may spread contiguously to involve deeper structures and underlying bone (Lipsky, 2014). A plethora of osteomyelitis (OM) research has illustrated the importance of several adhesins that facilitate microbial binding to bone matrix, particularly in context with *Staphylococcus aureus* (Dufrêne and Viljoen, 2020).

The acute presence of bacteria adhering to bone triggers a host immune response that has commonly been identified through histopathological, immunohistochemistry and flow cytometry studies *in vitro*, animal models and *in vivo* human studies. Characteristics in acute OM have demonstrated disruption to bone homeostasis (Josse et al., 2015), induction of the innate immune response (Hofstee et al., 2020) and hallmark features of clinical infection (Horst et al., 2012). This includes pattern recognition of conserved microbial antigens by toll like receptors (TLRs) present in osteal macrophages (Chang et al., 2008). Macrophage driven secretion of chemoattractants initiates infiltration of polymorphonuclear leukocytes (PMNs) from bone marrow and tissue, where they both contribute to the production of pro-inflammatory cytokines/chemokines, initiate phagocytosis, increase oxidative stress and produce antimicrobial peptides (Rigby and DeLeo, 2012; Lüthje et al., 2020). The adaptive immune response to pathogens in bone is marked by a cellular response in T lymphocytes and antibody responses mediated by B cells. Collectively, infection induced infiltrating PMN, and T cells contribute to induction of osteoclastogenesis, thus providing a link between inflammation and bone resorption (Gaida et al., 2012; Kumar et al., 2014).

The pathological presentation for diabetes related foot osteomyelitis (DFO) is suggestive of a more chronic, persistent mechanism of injury (Rao et al., 2011) and observations of acute OM or septic arthritis are less frequently observed. In cases where acute osteomyelitis is inadequately treated, pathological symptoms of a chronic nature can demonstrate abscess formation, remodeling of bone, increased bone fragility, altered vasculature, accelerated bone necrosis, fibrosis and formation of sequestrum (Wyzga et al., 2004; Cecilia-Matilla et al., 2013). Additionally, several alterations in T cell function, reduced PMN infiltration and bacterial evasion mechanisms suggest that Immune suppression plays a critical role in the development of chronic OM (Wang et al., 2017; Huang and Ge, 2020; Zoller et al., 2020).

We have previously demonstrated the presence of biofilms in chronic DFO as a potential cause of treatment failure (Johani et al., 2019; Malone et al., 2019), and recently adopted a multi-omics approach to better elucidate the host and microbe in skin and soft tissue diabetes foot infections (Radzieta et al., 2021). In this study we employed RNA-sequencing to explore microbial metatranscriptomes with a view to understand the relative activity and function of pathogen/s responsible for DFO.

## Results

Intra-operative bone specimens were collected from 25 individuals who required surgical intervention for management of their DFO, with bone specimens being divided in two for RNA sequencing (*n* = 25) and conventional culture (*n* = 20). Five bone specimens for conventional culture were not possible due to the small size of resected bone. Total-RNA sequencing generated a median of 142 million reads (±16 million reads) per sample. Following host depletion, a median of 38 million reads (±56 million reads) per sample were retained for microbial analysis using the SqueezeMeta pipeline. A PCA analysis of the taxonomic distribution of DFO wounds identified that most samples clustered relatively closely, with P40, P14, and P24 being outliers (Supplementary Figure 1).

**Figure 1:**
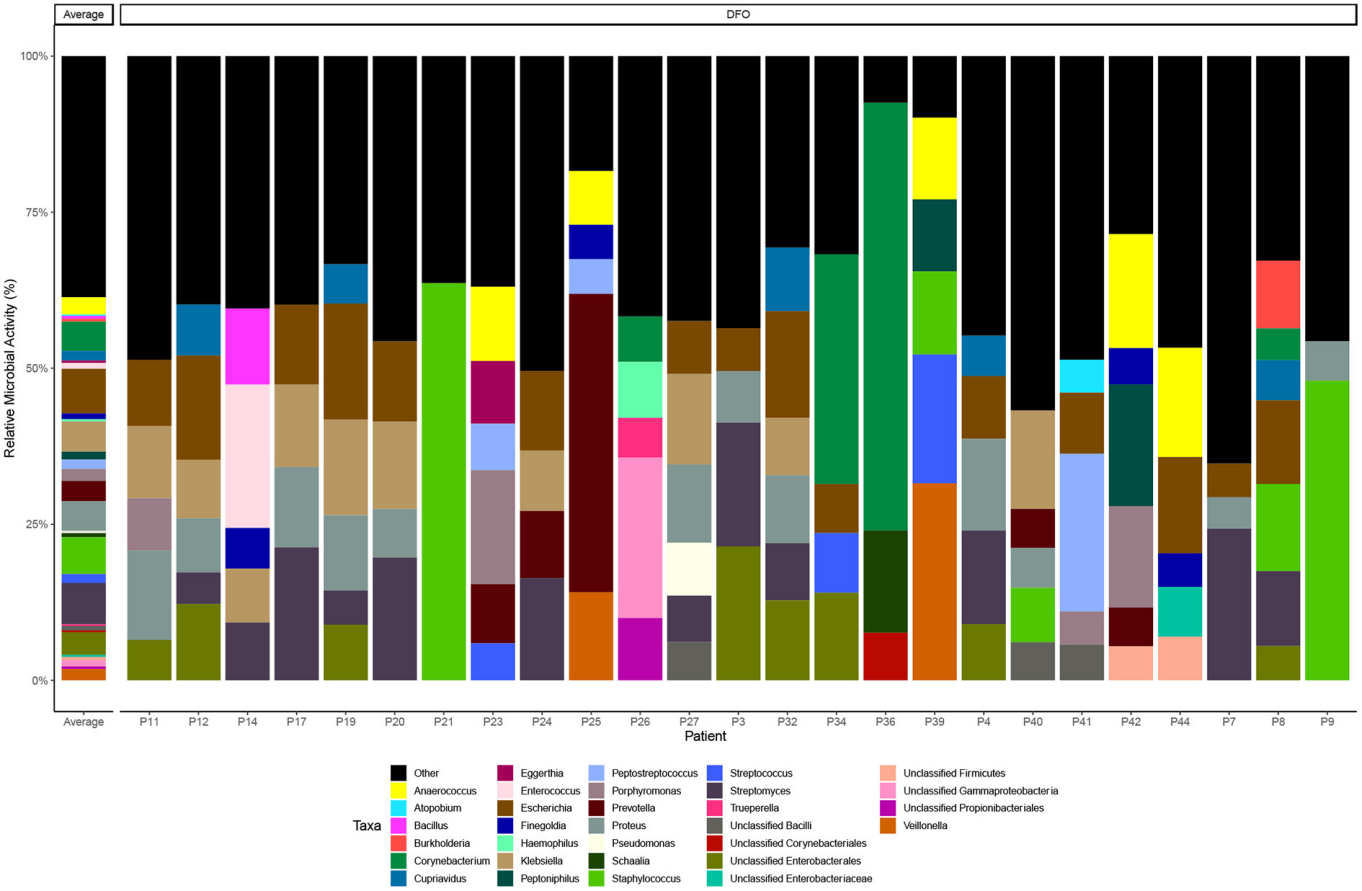
Taxonomic distribution of microbial metatranscriptomic data. Bar chart highlighting taxonomy at the genus level that represent > 5% of overall microbial activity. Taxa below the 5% threshold are grouped as “other”.

We then examined the relative microbial activity of DFO wounds stratified to genus level, observing that *Escherichia* spp. (7%), *Streptomyces* spp. (7%), *Staphylococcus* spp. (6%), *Klebsiella* spp. (5%) and *Proteus* spp. (5%) are the most active taxa on average across all DFO samples (Figure 1). However, in many cases the distribution of active taxa within DFOs was generally patient specific. For example, in P36 and P21 we observed relatively high homogeneity with the dominant taxa being *Corynebacterium* spp. and *Staphylococcus* spp., respectively. Conversely, other patients including P11, P12, P17, P19, P20, P24, P27, and P32 showed greater heterogeneity with higher proportions of *Escherichia* spp., *Klebsiella* spp. and *Proteus* spp.

Conventional culture data were also available for 20 bone samples (Supplementary material) revealing similar trends in the types of bacteria present; *Staphylococcus aureus* (*n* = 8, 40%), *Escherichia coli* (*n* = 6, 30%), *Streptococcus agalactiae* (*n* = 4, 20%), *Corynebacterium striatum* (*n* = 3, 15%), and *Pseudomonas aeruginosa* (*n* = 2, 10%). Similarly, like RNA-seq, the distribution of bacteria in bone specimens were generally specific to an individual and mostly non-concordant when comparing the isolates from culture vs. aligned taxonomic RNA transcripts. In a limited number of cases (*n* = 5) however we observed good concordance between culture and the presence of abundant *E. coli* RNA-transcripts.

**Figure 2:**
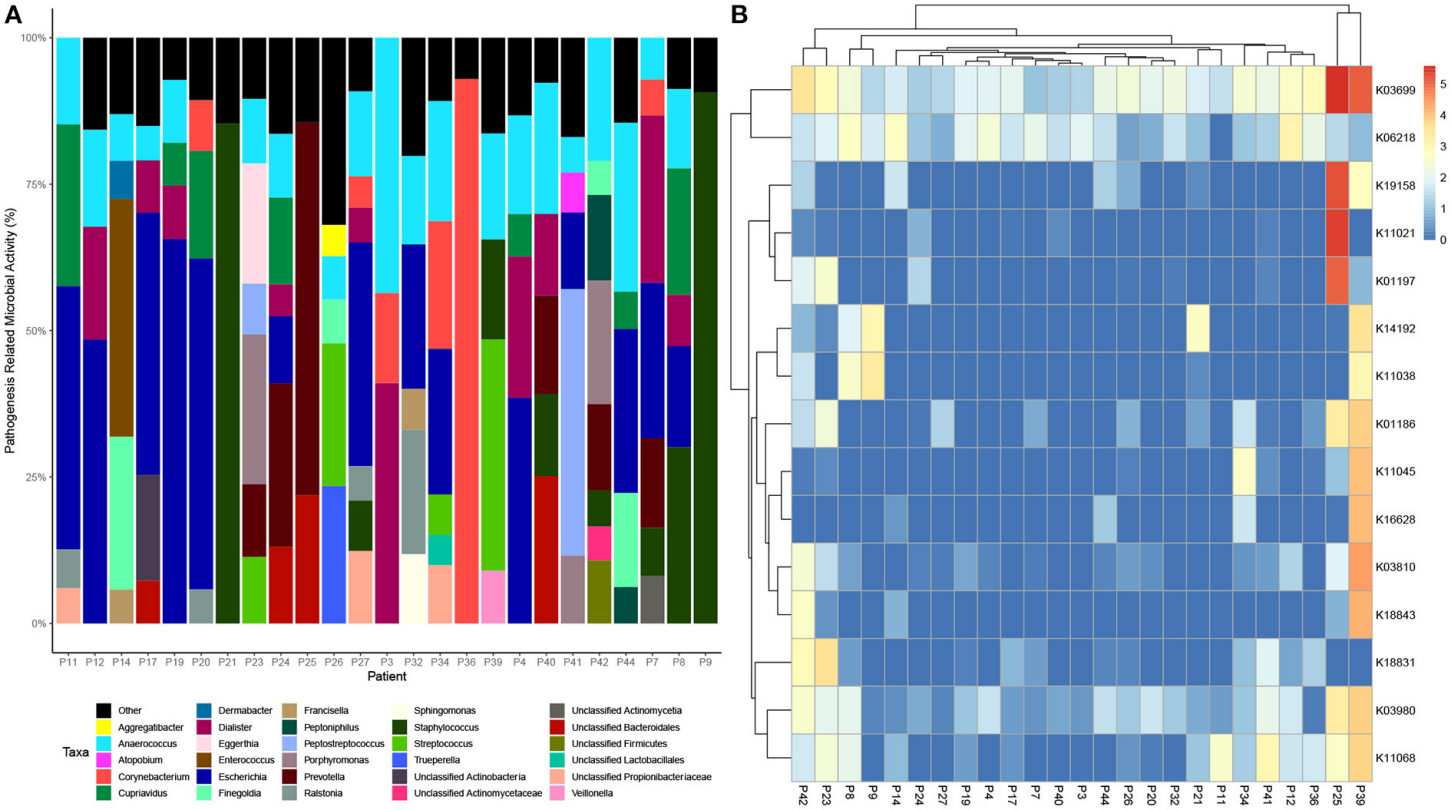
Taxonomic and functional distribution of metatranscriptomic data associated with pathogenesis. **(A)** Bar chart highlighting taxonomy at the genus level that represent > 5% of pathogenic activity. **(B)** Heatmap highlighting the top 15 expressed transcripts across all samples normalized to LogTPM.

**Figure 3:**
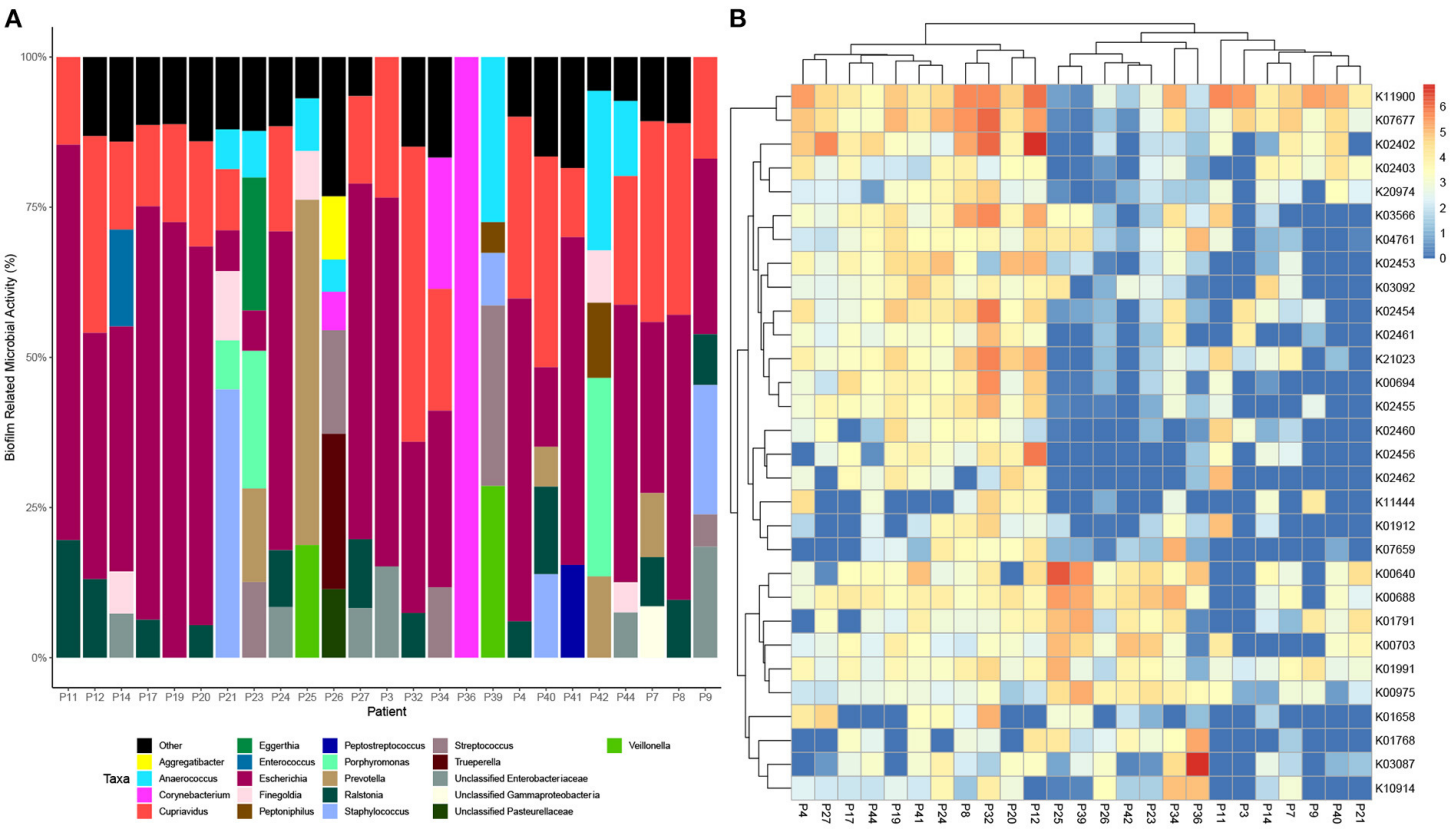
Taxonomic and functional distribution of metatranscriptomic data associated with biofilm. **(A)** Bar chart highlighting taxonomy at the genus level that represent > 5% of pathogenic activity. **(B)** Heatmap highlighting the top 30 expressed transcripts across all samples normalized to LogTPM.

**Figure 4:**
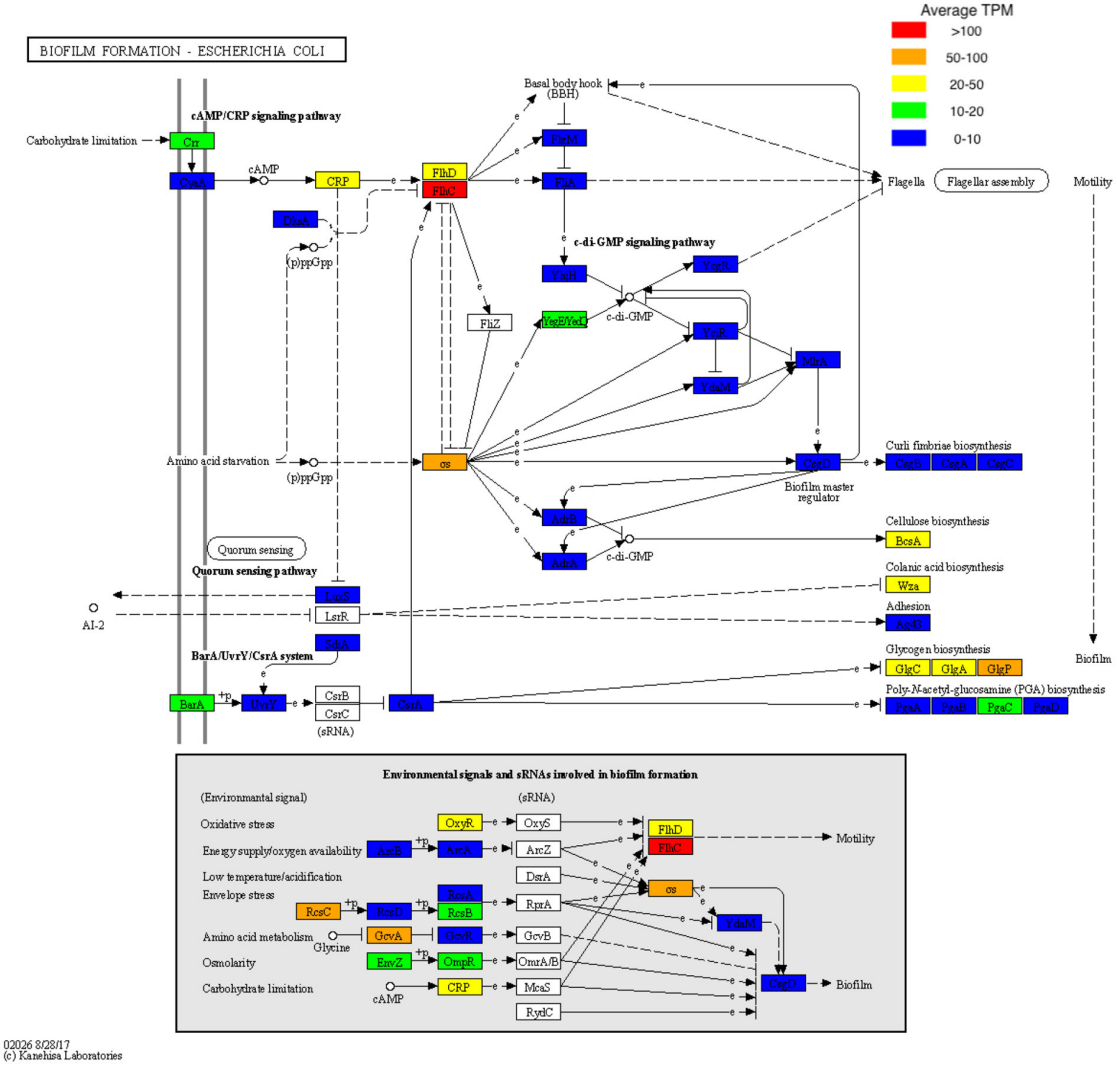
KEGG pathway map of pathway eco02026 with biofilm TPM data superimposed. TPM values were averaged across all samples and stratified based on TPM count prior to being visualized onto pathway eco02026.

Data was then subset to examine functions associated with pathogenesis (virulence and toxins), biofilm formation and antimicrobial resistance (LogTPM). Analysis revealed *Escherichia* spp. (19%), *Anaerococcus* spp. (12%) and *Staphylococcus* spp. (10%) are the most active taxa relative to pathogenic functions (Figure 2A), with K06218 (mRNA interferase *relE*), K03699 (membrane damaging toxin *tlyC*) and K03980 (putative peptidoglycan lipid II flippase *murJ*), K01114 (membrane damaging toxin plc) and K19168 (toxin *cptA*) being the most prevalent pathogenic associated transcripts (Figure 2B; Table 1). Sub-setting for biofilm associated functions revealed that *Escherichia* spp. (35%) were the dominant taxa across DFO samples, followed by *Cupriavidus* spp. (17%), *Ralstonia* spp. (5%) and *Corynebacterium* spp. (5%) (Figure 3A). Functional analysis identified numerous biofilms associated transcripts within DFO wounds (Figure 3B). Across all patients, the most abundant transcripts included those associated with components of the biofilm EPS matrix including glycogen synthesis, cellulose synthesis, colonic acid synthesis and flagella synthesis (Figure 4).

**Table 1:** Top expressed transcripts associated with pathogenesis.

**KEGG ID**	**Gene**	**Function**	**Percentage of DFO samples**
K06218	*relE*	mRNA interferase	96%
K03699	*tlyC*	Magnesium and cobalt exporter/membrane damaging toxin	100%
K03980	*murJ*	Peptidoglycan lipid II flippase	100%
K19165	*phd*	Antitoxin Phd	52%
K09159	*cptB*	Antitoxin CptB	40%
K01114	*plc*	Membrane damaging toxin	72%
K19168	*cptA*	Toxin CptA	72%
K21498	*higA-1*	Antitoxin higA-1	48%
K11068	*hlyIII*	Hemolysin III	64%
K18831	*higA*	Antitoxin HigA	48%
K07334	*higB-1*	Toxin HigB-1	48%
K03810	*mviM*	Virulence factor	68%
K01186	*NA*	NA	NA
K01197	*hya*	Collagenase toxin	48%
K07473	*dinJ*	DNA damage inducible protein	36%

**Figure 5:**
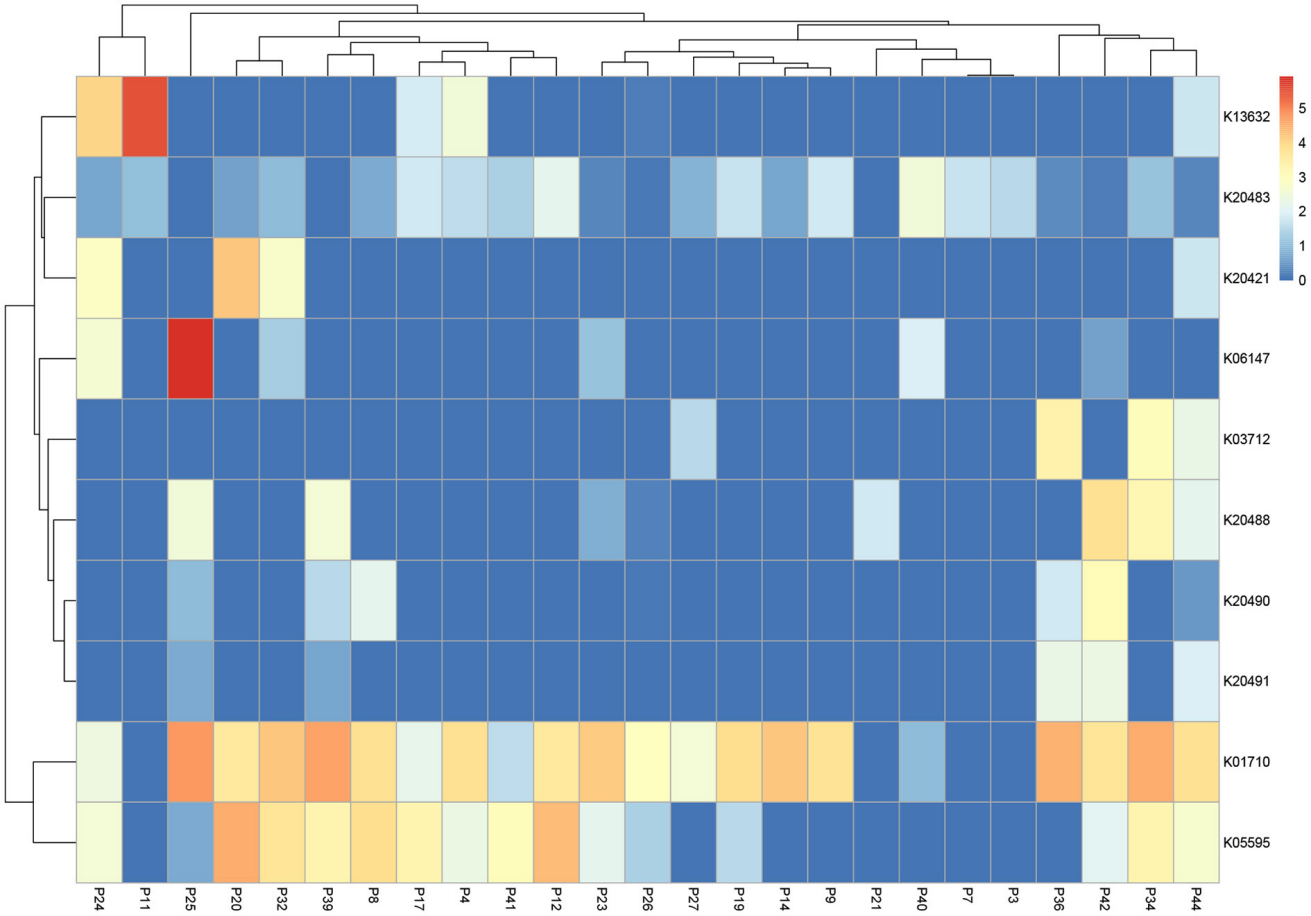
Heatmap highlighting the top 10 expressed transcripts across all samples associated with “antibiotics and multi-drug resistance”. Data is normalized and displayed to LogTPM.

We also explored functions associated with antibiotic and multidrug resistance reporting the top ten most abundant transcripts (Figure 5). Analysis revealed enrichment of K01710 (dTDP-glucose 4,6-dehydratase *rfbB, rmlB, rffG*) present in 21 of 25 bone samples, K20483 (lantibiotic biosynthesis protein *nisB*) in 17 of 25 bone samples and K05595 (multiple antibiotic resistance protein *marC*) in 16 of 25 bone samples.

## Discussion

The microbiome associated with DFO has been reported in the literature several times in recent years based on targeted 16S sequencing data (van Asten et al., 2016; Johani et al., 2019; Malone et al., 2019). However, these studies suffer from the shortfall of 16S sequencing by their inability to perform functional analysis on bacteria present. In this study, we utilized a metatranscriptomic approach to examine which bacteria are metabolically active within DFO using total-RNA sequencing data. Analysis of the DFO metatranscriptome revealed an alternate view of the microorganisms demonstrating high relative activity compared to previous studies reporting culture isolates of DFO pathogens using conventional culture methods or the relative abundance using 16S sequencing (Senneville et al., 2006; van Asten et al., 2016; Malone et al., 2019).

Studies utilizing 16S sequencing have identified *Corynebacterium* spp., *Finegoldi*a spp., *Staphylococcus* spp., *Streptococcus* spp., *Porphyromonas* spp., and *Anaerococcus* spp. as the most abundant within DFOs (van Asten et al., 2016; Malone et al., 2019). Studies employing conventional culture have commonly reported aerobic Gram-positive cocci (*Staphylococcus aureus* and *Streptococcus* sp.) as the predominant pathogens in DFO, with Gram-negative bacilli being reported to a much lesser degree (Richard et al., 2011; Uçkay et al., 2015). In this study *Escherichia* spp. and *Klebsiella* spp. demonstrated a high relative microbial activity across most DFO samples (*n* = 15). In contrast, *Staphylococcus* spp. demonstrated high relative microbial activity in four DFO samples, with *Streptococcus* spp. and *Corynebacterium* spp. in three DFO samples.

Furthermore, when filtering RNA transcripts for functions associated with pathogenesis (virulence and toxin production) and biofilm, *Escherichia* spp. was observed as being the predominant microorganism, contributing to 19 and 35% of total mean RNA transcripts across all patients, respectively. *Escherichia* spp. are not commonly reported in culture data for DFO, however a study by Uckay et al. identified that *P. aeruginosa* and *Enterobacteriaceae* are considerably more dominant within subtropical regions (Uçkay et al., 2015). Recently, Lienard et al. examined the clonal diversity of *E. coli* strains isolated from skin and soft tissue diabetes foot infections (DFI) and DFO over a two-year period. Lienard et al. (2021) reported that *E. coli* is readily capable of adapting to stressful conditions in bone cells through genome reduction, metabolic modifications and through the balancing of virulence determinants.

In this study, conventional culture from intraoperative bone specimens (*n* = 20) identified *S. aureus* (*n* = 8) as the predominant isolate, with the second most reported isolate *E. coli* (*n* = 6), followed by *S. agalactiae* (*n* = 4) and *Corynebacterium striatum* (*n* = 3). All patients in this study received a period of conservative medical management (with antibiotic therapy) in an out-patient setting, but ultimately required surgical intervention to resolve the DFO. Initial antibiotic therapy for suspected DFO was guided using tissue biopsies or deep wound swabs from diabetic related foot ulcers (DRFUs), as percutaneous bone biopsy was not available for patients enrolled in this study.

The most common oral antibiotics prescribed before the inclusion of patients were amoxicillin and clavulanic acid (*n* = 8), flucloxacillin or dicloxacillin (*n* = 5), clindamycin (*n* = 3), cephalexin (*n* = 2), ciprofloxacin (*n* = 2) and trimethoprim/sulfamethoxazole (*n* = 2). Out-patient based parenteral therapy included cefazolin (*n* = 3) and ceftriaxone (*n* = 1). Prescribing practices were based on the Therapeutic Guidelines for Australia (Therapeutic Guidelines, 2021) for the management of mild to moderate DFIs in patients with chronic ulceration who may have received previous antibiotic therapy. In context to our findings some of the antibiotic regimens prescribed would provide sufficient coverage against *Escherichia* spp., with cephalosporins, fluoroquinolones, and trimethoprim-sulfamethoxazole being considered as first line therapies. Oral amoxicillin/clavulanate was the most prescribed antibiotic in this study and is routinely prescribed for respiratory tract infections, skin infections as well as urinary tract infections (UTIs). In the context of rising extended spectrum beta-lactamase (ESBL) prevalence globally, empirical and overuse of orally administered amoxicillin/clavulanate may select resistance in Gram-negative pathogens (Veeraraghavan et al., 2021).

Non-surgical management may have been improved if percutaneous bone biopsy was performed as this provides more accurate culture data allowing for targeted antibiotic therapy (Senneville et al., 2006). This could account for the failure to achieve infection resolution with non-surgical management in this cohort, but it may not be the only contributing factor. Previous work by our group has identified that biofilms likely contribute to the chronic infections of DFO, commonly observing rod-shaped bacteria using microscopy techniques (Malone et al., 2019). Our findings in this study support the premise of biofilm being a potential driver in DFO, with genes associated with cellulose synthesis, colonic acid synthesis, glycogen synthesis and flagella synthesis being the most highly expressed on average within DFO. Respectively, these processes are essential for the structural integrity of biofilms, facilitating the expansion of voluminous biofilms and the initiation of biofilm formation (Bonafonte et al., 2000; Belas, 2014).

Analysis of pathogenicity factors identified *ytfL*, and *relE* transcripts within the majority of DFO samples. Iwadate et al. reported that *ytfL* expression facilitates cell viability of *E. coli* and *S. typhirium* when exposed to polyamines such as putrescine and cadaverine, both of which are abundant within necrotic diabetes related foot ulcers (Iwadate et al., 2021). Previous studies have reported that *relE* overexpression results in reduced bacterial growth and increased persister cell formation in *Xylella fastidiosa* and *E. coli* (Tashiro et al., 2012; Burbank and Stenger, 2017). Therefore, the expression of *relE* observed in 24/25 patients may be indicative of increased abundance of persister/dormant cells within DFOs. Persister cells are dormant phenotypic variants with increased antibiotic tolerance, found within a susceptible bacterial population and could account for high conservative management (antibiotic) failures in DFO (Gimza and Cassat, 2021).

We explored genes broadly associated with the key word “antibiotics”. K01710 (dTDP-glucose 4,6-dehydratase *rfbB, rmlB, rffG*) was abundant across all but four bone specimens. dTDP-D-glucose 4,6-dehydratase is an enzyme which catalyzes the dehydration of the nucleotide sugar dTDP- D-glucose (Vogel et al., 2022). L-rhamnose is found in the cell walls and envelopes of many pathogenic Gram-negative and some Gram-positive bacteria (Mäki and Renkonen, 2004), and the enzyme is an important constituent of the L-rhamnose biosynthetic pathway which is involved in a variety of biological functions including bacterial fitness (Solheim et al., 2014; Koller and Lassak, 2021) growth (van der Beek et al., 2019), virulence (Allard et al., 2002; Zulianello et al., 2006), extracellular polysaccharide (EPS) (Whitfield and Paiment, 2003; Mistou et al., 2016), and lipopolysaccharide (LPS) production (King et al., 2009; Franzosa et al., 2018). L-rhamnose biosynthesis enzymes are not encoded by mammalian cells and have therefore been proposed as targets for novel antibiotic drugs (Allard et al., 2002), a proposal which is indirectly supported by the findings of our study.

## Conclusion

*Escherichia* is not commonly reported as a pathogen of DFO. In this study, RNA-sequencing reveals *Escherichia* spp., as the most predominant microorganism in context to the relative activity of functions associated with pathogenesis (virulence and toxin production) and biofilm pathways. We have previously reported on the role of biofilms as drivers of chronic infections in DFO, potentially contributing to antibiotic tolerance and treatment failure (Johani et al., 2019). Our findings may also have potential clinical implications with regards to the antibiotic regimens used for the non-surgical management of DFO. Most of the antibiotic regimens used in this study prior to surgical intervention would provide insufficient coverage against *Escherichia* spp., with cephalosporins, fluoroquinolones, and trimethoprim-sulfamethoxazole being considered as first line therapies. The results from this study could be reflective of persons who fail non-surgical management of their DFO, and this may be different from persons who experience successful resolution of infection. Future research should therefore include and compare both cohorts, in addition to obtaining bone specimens percutaneously through healthy skin. Currently, there is also lack of evidence with regards to the types of antibiotics used in the non-surgical management of DFO, and/or if certain antibiotics are superior to others. Research in this area would be of great value in improving non-surgical management outcomes for DFO.

## Methods

### Study design

Over a 12-month period, we prospectively enrolled 25 consecutive persons aged over 18 years who presented to the Liverpool Hospital Vascular Surgery Department (Table 2). Subjects were eligible for the study if there was a high clinical suspicion of underlying or confirmed DFO. Treating clinicians used the international working group on diabetic foot (IWGDF) PEDIS classification scheme (Lipsky et al., 2020) as a guide to the clinical likelihood of the presence of DFO, based on available clinical, radiological and laboratory data. Patient demographics, laboratory and clinical data were collected through patient charts and electronic medical records. Radiological evidence of DFO was routinely sought on plain X-rays (appearance of cortical erosion, lucencies in cortex, bone resorption, fragmentation or sequestrate); in selected cases patients were assessed by single photon emission computed tomography (SPEC/CT) or magnetic resonance imaging (MRI). In patients with clinically and radiologically suspected DFO, we confirmed its presence as a positive culture and/or molecular (DNA sequencing) test of aseptically collected bone (Senneville et al., 2006). Exclusion criteria for this study included: patients requiring revision surgery of previously diagnosed DFO; the presence of gangrene or necrotising soft tissue infection; or evidence of Charcot neuropathic osteoarthropathy.

**Table 2:** Key resources table.

**Reagent or resource**	**Source**	**Identifier**
**Biological samples**		
Diabetic foot osteomyelitis—bone specimens	Human *in vivo*	See metadata.csv in supplement for full metadata
**Critical commercial assays**		
QubitTM dsDNA HS Assay Kit	Life Technologies	Cat#Q32854
Zymo Host Zero Microbial DNA Kit	Zymo	Cat#D4310
TRIzol plus total transcriptome isolation kit	ThermoFisher	Cat#12183555
**Deposited data**		
Total RNA sequencing data	This paper	Sequence Read Archive (SRA)/NCBI (http://www.ncbi.nlm.nih.gov/sra) accession number PRJNA832884.
**Software and algorithms**		
HUMAnN2	Franzosa et al. (2018)	http://huttenhower.sph.harvard.edu/humann
Bowtie2	Langmead and Salzberg (2012)	http://bowtie-bio.sourceforge.net/bowtie2/index.shtml
BBTools	Bushnell et al. (2017)	https://sourceforge.net/projects/bbmap/
Trim Galore	Martin (2011)	https://github.com/FelixKrueger/TrimGalore
STAR	Dobin et al. (2013)	https://github.com/alexdobin/STAR
RSEM	Li and Dewey (2011)	https://github.com/deweylab/RSEM
EdgeR	Robinson et al. (2010)	https://bioconductor.org/packages/edgeR/
SqueezeMeta	Tamames and Puente-Sanchez (2019)	https://github.com/jtamames/SqueezeMeta
GenBank	Clark et al. (2016)	https://www.ncbi.nlm.nih.gov/genbank/
KEGG	Kanehisa and Goto (2000)	https://www.genome.jp/kegg/

Surgeons collected intra-operative bone specimens from persons who required surgical intervention (resection or amputation) for management of their DFO. Following surgical debridement of infected or necrotic tissue, bone sections were visually inspected by the surgeon and correlated against pre-operative imaging. Suspected infected bone margins were harvested using new instruments, either bone nibblers or an electric pen drive oscillating saw. All bone specimens were immediately placed in to RNA*later* (Thermo Fisher Scientific, Waltham, MA, United States) stabilization solution for 24 h at 4°C and then stored at −80°C until processed.

Permission to conduct the study was granted by the South Western Sydney Local Health District Research and Ethics Committee (HREC/16/LPOOL/475, SSA/16/LPOOL/476). The study methodology was designed in accord with, and our molecular surveillance data are reported in keeping with, the “Strengthening the Reporting of Molecular Epidemiology for Infectious Diseases (STROME-ID-STROBE)” statement (Field et al., 2014).

## RNA isolation and library preparation

Bone samples were frozen in liquid nitrogen then immediately crushed in a TissueLyser II using a 7 mm stainless steel ball. The homogenized sample was then transferred to a tube containing a mixture of 0.5 and 2 mm zirconia beads with Trizol and homogenized again. Chloroform was then added, and the sample was centrifuged to isolate the RNA containing aqueous phase. Isolated RNA was precipitated using isoporopanol then washed twice with 70% ethanol prior to library preparation. Ribodepletion and library construction were then performed using the Zymo-Seq RiboFree total RNA kit (Zymo Research, CA, USA) prior to sequencing on the Novaseq 6000 S4 platform (Illumina, CA, USA) at 2 × 150 bp to ensure a sufficient output of reads per sample.

## Culture-dependent bacteriological enumeration and identification

Culture-dependent analysis of tissue cultures was performed by a hospital microbiology service (Sydney South West Pathology Service). Briefly, bone specimens were weighed and homogenized using a sterile tissue pulper in 3 ml of sterile saline. Plates were streaked for isolation onto four quadrants of recommended agars and grown under appropriate atmospheres to isolate clinically relevant organisms (both aerobe and anaerobe) per standardized methods. Bacterial identification was performed using matrix-assisted laser desorption ionization-time of flight mass spectrometry (MALDI-TOF MS).

## Processing of metatranscriptome data

RNA-seq generated approximately × reads (±20 SEM) per sample with a mean ribosomal content of %. Reads were trimmed using TrimGalore/Cutadapt (Martin, 2011) and aligned in paired-end mode to GRCh38.p12 with alternative haplotypes and unlocalised contigs removed, using STAR2.5.4b (Dobin et al., 2013). Following host filtering, microbial analysis was completed using the SqueezeMeta pipeline (v1.5) (Tamames and Puente-Sanchez, 2019) utilizing the co-assembly option with no binning and doublepass mapping enabled. Briefly, paired end reads were assembled using RNASpades prior to taxonomic and functional annotation using the DIAMOND sequencing aligner to the GenBank and KEGG databases, respectively. Reads of individual samples were then be mapped to assembled contigs for the estimation of taxonomic and functional abundances using Bowtie2. Relative activity plots for each DFO phenotype was then generated using R, based on the raw read counts mapping to each taxa. Read counts were normalized (LogCPM), and a principal coordinate analysis (PCA) was performed to demonstrate any variation among datasets based on their taxonomic and functional profiles. Functional analysis was completed using normalized (LogTPM) counts output from the SqueezeMeta pipeline.

## Quantification and statistical analysis

The R Statistical Package (R Core Team, 2017) was used to generate all figures and compute statistical analysis.

## Data availability statement

The datasets presented in this study can be found in online repositories. The names of the repository/repositories and accession number(s) can be found in the article/Supplementary material.

## Ethics statement

Permission to conduct the study was granted by the South Western Sydney Local Health District Research and Ethics Committee (HREC/16/LPOOL/475 and SSA/16/LPOOL/476). The patients/participants provided their written informed consent to participate in this study.

## Author contributions

MM: conceptualization. MM and MR: methodology, formal analysis, and writing—original draft. MM and SS: recruitment of patients and sample collection. MM, MR, and HD: writing—review and editing. MM: funding acquisition. All authors contributed to the article and approved the submitted version.

## Conflict of interest

The authors declare that the research was conducted in the absence of any commercial or financial relationships that could be construed as a potential conflict of interest.

## Publisher's note

All claims expressed in this article are solely those of the authors and do not necessarily represent those of their affiliated organizations, or those of the publisher, the editors and the reviewers. Any product that may be evaluated in this article, or claim that may be made by its manufacturer, is not guaranteed or endorsed by the publisher.
