# Supplementary material for: Metatranscriptome sequencing identifies Escherichia are major contributors to pathogenic functions and biofilm formation in diabetes related foot osteomyelitis
Source: Front Microbiol. 2022 Aug 1;13:956332. doi: 10.3389/fmicb.2022.956332 (PMC9376677; doi:10.3389/fmicb.2022.956332)
Supplement: Supplementary file 1 [file Data_Sheet_1.docx]

**Supplementary information**

Supplementary Figure 1: PCA of microbial taxonomic transcripts across all samples

Supplementary Figure 2: PCA of functional transcripts across all samples
